# Repeated clinical malaria episodes are associated with modification of the immune system in children

**DOI:** 10.1186/s12916-019-1292-y

**Published:** 2019-03-13

**Authors:** Yaw Bediako, Rhys Adams, Adam J. Reid, John Joseph Valletta, Francis M. Ndungu, Jan Sodenkamp, Jedidah Mwacharo, Joyce Mwongeli Ngoi, Domtila Kimani, Oscar Kai, Juliana Wambua, George Nyangweso, Etienne P. de Villiers, Mandy Sanders, Magda Ewa Lotkowska, Jing-Wen Lin, Sarah Manni, John W. G. Addy, Mario Recker, Chris Newbold, Matthew Berriman, Philip Bejon, Kevin Marsh, Jean Langhorne

**Affiliations:** 1 Francis Crick Institute, London, UK; 2 Wellcome Genome Campus, Wellcome Sanger Institute, Hinxton, Cambridgeshire, UK; 3 University of Exeter, Exeter, UK; 4 KEMRI/Wellcome Trust Research Programme, Kilifi, Kenya; 5 Weatherall Institute of Molecular Medicine, University of Oxford, Oxford, UK; 6 Nuffield Department of Medicine, University of Oxford, Oxford, UK; 7 Present Address: Transla TUM, Zentralinstitut für translationale Krebsforschung der Technischen Universität München, Munich, Germany; 8 Present Address: West African Centre for Cell Biology of Infectious Pathogens, University of Ghana, Accra, Ghana; 9 Present Address: Division of Pediatric Infectious Diseases, State Key Laboratory of Biotherapy, Sichuan University and Collaboration Innovation Centre, Chengdu, China

**Keywords:** Malaria, Systems immunology, Immune activation

## Abstract

**Background:**

There are over 200 million reported cases of malaria each year, and most children living in endemic areas will experience multiple episodes of clinical disease before puberty. We set out to understand how frequent clinical malaria, which elicits a strong inflammatory response, affects the immune system and whether these modifications are observable in the absence of detectable parasitaemia.

**Methods:**

We used a multi-dimensional approach comprising whole blood transcriptomic, cellular and plasma cytokine analyses on a cohort of children living with endemic malaria, but uninfected at sampling, who had been under active surveillance for malaria for 8 years. Children were categorised into two groups depending on the cumulative number of episodes experienced: high (≥ 8) or low (< 5).

**Results:**

We observe that multiple episodes of malaria are associated with modification of the immune system. Children who had experienced a large number of episodes demonstrated upregulation of interferon-inducible genes, a clear increase in circulating levels of the immunoregulatory cytokine IL-10 and enhanced activation of neutrophils, B cells and CD8^+^ T cells.

**Conclusion:**

Transcriptomic analysis together with cytokine and immune cell profiling of peripheral blood can robustly detect immune differences between children with different numbers of prior malaria episodes. Multiple episodes of malaria are associated with modification of the immune system in children. Such immune modifications may have implications for the initiation of subsequent immune responses and the induction of vaccine-mediated protection.

**Electronic supplementary material:**

The online version of this article (10.1186/s12916-019-1292-y) contains supplementary material, which is available to authorized users.

## Background

Malaria is caused by infection with the protozoan parasite *Plasmodium* spp. and is responsible for approximately half a million deaths annually. Most of the mortality occurs among children under 5 years of age [1], and progress in control has recently stalled [2]. Malaria pathogenesis is characterised by a complex interplay between an antigenically diverse parasite and a constantly evolving immune response in the host. Initial exposure often leads to disease, but subsequent repeated exposures lead to the development of partially protective, non-sterile immunity [3–5]. There is mounting evidence that repeated clinical episodes of malaria result in substantial modification of the host immune system. *P. falciparum* (*Pf*) infection has been shown to stimulate T regulatory cells [6, 7] and to significantly alter the phenotype and function of a number of other immune cell populations including dendritic cells [8], conventional B [9, 10] and T lymphocytes [11, 12] and γδ T cells [13]. In line with this, some *Pf* proteins bind the inhibitory receptor LILRB1 found on NK and B cells [14].

The consequences of such immune modification have not been studied extensively; however, it is interesting to note that a number of vaccine candidates have demonstrated much-reduced efficacy when tested in malaria-endemic populations as compared to malaria-naïve populations [15, 16]. Although the precise mechanism of this is not fully understood, it suggests that complex interactions between malaria and the immune system affect the ability to elicit appropriate immune responses upon challenge. Whether such immune modification persists in the absence of parasitaemia (steady state) is also not known.

Here, we examined healthy uninfected children living in an endemic area who had been under active surveillance for clinical malaria for 8 years and had experienced either high or low numbers of clinical episodes (relative to the population average). We took a multi-dimensional approach, comprising whole blood transcriptomic, cellular and plasma cytokine analyses to describe the immune systems in these two groups of children, providing a comprehensive description of the effect of repeated episodes of clinical malaria on the steady-state immune system of children living in an endemic area. While insufficient to establish the causal relationship between malaria episodes and any immune modification (differences could reflect inherent immunological differences that predispose certain individuals to increased numbers of episodes), this study represents a necessary first step in furthering our understanding of the complexity of malaria immune responses.

## Materials and methods

### Study population

The participants for this study were drawn from two previously described cohorts of children who had been under active weekly surveillance for 8 years [17, 18]. The Junju cohort is in an area of moderate malaria transmission with a *Pf* prevalence of approximately 30% [15, 17] during the rainy season, while the Ngerenya cohort is in an area where malaria transmission has fallen and remained at almost zero since 2004 [18]. As described elsewhere [19, 20], children were visited every week by field workers (themselves living within the local community) for the detection of malaria-associated fevers and who were also available to assess any fevers occurring between weekly visits. Any child with an axillary body temperature of greater than 37.5 °C was tested for *Pf* parasitaemia by rapid diagnostic test and confirmed by microscopic examination of thin and thick blood smears stained with 10% Giemsa. A clinical episode of malaria was defined as body temperature above 37.5 °C with > 2500 parasites per microlitre of blood.

For our analysis, 42 children of similar age (7–10.5 years) were selected belonging to 2 categories—“low” and “high” (under active surveillance since 2007) depending on their number of past clinical episodes. An additional 27 age-matched children who had never had clinical malaria (naïve) were selected from Ngerenya (under active surveillance since 1989), where malaria transmission has remained very low since 2004. The low group consisted of children from Junju who had less than 5 recorded episodes of malaria, while the high group (also selected from Junju) had between 8 and 18 recorded episodes of malaria. A single blood sample was taken from each child and processed as described below. All 69 children were genotyped to confirm that none carried the sickle cell trait (haemoglobin AS genotype), a well-characterised polymorphism associated with resistance to malaria infection [21]. All 69 children were also determined to be negative for *Pf* (microscopy and PCR) and had not had a clinical episode within the last 110 days prior to sampling.

### Sample collection

Five millilitres of blood was drawn from each child by venesection in March 2015 prior to the start of the major malaria transmission season. One millilitre was immediately placed in a Tempus tube (Thermo Fisher Scientific) and stored for downstream transcriptomic analysis. The remaining blood was transported within 2 h of collection to the laboratory where 200 μL was aliquoted for flow cytometry and 100 μL aliquoted for real-time PCR (to assess *Pf* status), and the remaining sample was centrifuged to separate the plasma which was stored at − 20 °C.

### PCR analysis

For PCR analysis, DNA was first extracted from 30 μL of whole blood using QIAxtractor machine (QIAGEN, Hilden, Germany). The DNA was eluted in 100 μL, from which 5 μL of DNA were amplified by quantitative PCR. This was done using a TaqMan assay for the *Pf* multicopy 18S ribosomal RNA genes, as described elsewhere [22], except that we used a modified probe (5′-FAM-AACAATTGGAGGGCAAG-NFQ-MGB-3′). We used an Applied Biosystems 7500 Real-Time PCR System with quantification by Applied Biosystems 7500 software v2.0.6. Samples were analysed in singlet wells. Three negative control wells and 7 serial dilutions of DNA extracted from in vitro parasite cultures were included as standards on each plate in triplicate. Plates failing quality control standards were repeated. The lower limit of accurate quantification of this method is 10 parasites/mL within the PCR elute. By assessing 1/20 of 30 μL of blood with a gene target present on 3 chromosomes, the method has a theoretical limitation of 4.5 parasites/μL of whole blood, compared with a sensitivity of 50 parasites/μL for thick blood films. PCR standards were monitored through internal quality assurance and use of external quality control standards.

### Stool microscopy

The formol-ether concentration method was used to prepare samples for the detection of helminths or their eggs by microscopy.

### Flow cytometry

Two hundred microlitres of whole blood was mixed with a cocktail of monoclonal antibodies specific for human immune cell surface markers. The cocktail consisted of antibodies against CD3, CD4, CD8, CD14, CD16, HLA-DR, CD11c, CD45RO, CD45RA, TCR γδ, CD56, CD19 and CD303 as well as a live/dead stain (see Additional file 1: Table S1 for antibody conjugation information). After staining for 30 min at 4 °C, erythrocytes were lysed using BD FACS Lysing Solution (BD Biosciences, San Jose, CA). Cells were washed and re-suspended in 200 μL of 1× PBS and analysed on a BD Fortessa flow cytometer (BD Biosciences, San Jose, CA) acquiring at least 200,000 leukocyte events per sample. Given the size of the study and the need to limit time between sample collection and FACS analysis, sample collection and FACS were performed in batches over a number of days, with appropriate single-colour controls acquired on each day. All FACS data were however analysed together once all the samples had been collected. Initial compensation and manual gating analysis were performed using FlowJo (FlowJo LLC, Ashland, OR).

### Unsupervised FACS analysis

Flow cytometry data was analysed using the integrated analysis pipeline Cytofkit, available as an open-source R/Bioconductor package [23]. Briefly, fcs files containing all live gated, singlet events from each participant were imported, the expression values of each marker extracted from each fcs file and the extracted data transformed using “automatic logicle transformation”. Expression matrices from all fcs files were then combined into a single matrix, by sampling up to 10,000 events from each fcs file. Dimensionality reduction was performed using the Barnes-Hut variant of the t-SNE algorithm [24], and cellular subsets were identified using the clustering method proposed by Rodriguez and Laio [25]. Individual clusters were then manually annotated using a heatmap displaying the median intensity values per cluster for every marker. This heatmap was used to identify each cluster’s defining markers and designate each cluster as a previously described population or unknown population. For each cellular population, we performed a Kruskal-Wallis test between the three groups of children. For significant cell types, we performed a post-hoc Dunn’s test between each group.

### Plasma cytokine analysis

One hundred microlitres of plasma from each participant was submitted to Eve Technologies (Calgary, Canada) for analysis using the Human Cytokine/Chemokine 65-plex Discovery Assay. This multiplex assay is based on the Millipore MILLIPLEX cytokine array and is designed to detect and quantify the levels of the following cytokines: EGF, eotaxin, FGF-2, Flt-3 ligand, fractalkine, G-CSF, GM-CSF, GRO, IFN-α2, IFN-γ, IL-10, IL-12 (p40), IL-12 (p70), IL-13, IL-15, IL-17A, IL-1ra, IL-1α, IL-1β, IL-2, IL-3, IL-4, IL-5, IL-6, IL-7, IL-8, IL-9, IP-10, MCP-1, MCP-3, MDC (CCL22), MIP-1α, MIP-1β, PDGF-AA, PDGF-AB/BB, RANTES, TGFα, TNF-α, TNF-β, VEGF, sCD40L, Eotaxin-2, MCP-2, BCA-1, MCP-4, I-309, IL-16, TARC, 6CKine, eotaxin-3, LIF, TPO, SCF, TSLP, IL-33, IL-20, IL-21, IL-23, TRAIL, CTACK, SDF-1α+β, ENA-78, MIP-1d and IL-28A. Cytokine levels were parameterised as log fluorescence and tested using a three-way Kruskal-Wallis test between the naive, low-episode and high-episode groups. Post-hoc Dunn’s tests were performed on cytokines with significant differences.

#### RNA isolation and library preparation

Tempus/blood mix (1 mL blood with 6 mL Tempus solution) was thawed on ice for 1 h and transferred into a 50-mL Falcon tube. Next, 2 mL of ice cold 1×PBS was added to the samples followed by the addition of 3 mL chilled 100% ethanol. Samples were immediately vortexed for 30 s and then spun down at 15,000 rcf for 60 min at 0 °C. After centrifugation, the supernatant was removed and the emptied tubes blotted on clean absorbent paper to remove the remaining foam. No cell debris pellet was visible within the tube. Next, the cells were lysed by adding 200 μL of freshly prepared lysis/TCEP solution (Perfect Pure kit, 5’PRIME) to the pellet and vortexed immediately for 1.5 min. RNA isolation was performed using the Perfect Pure kit, following the manufacturer’s instructions, and eluted in 40 μL of nuclease-free water. Globin mRNA was depleted from the total RNA using the GLOBINclear kit (Ambion). Indexed libraries were then generated using the KAPA Stranded mRNA-Seq Kit (Roche) on an automated platform with 10 cycles of PCR amplification.

#### RNA sequencing

Seventy-five samples, comprising 6 replicates of a single European sample (batch controls), 27 samples from naive children and 42 samples from exposed children, were sequenced in a single multiplexed pool using 5 lanes (75 bp PE) of a HiSeq 2500 (Illumina). The reads were combined across lanes for each sample but not across runs and mapped using Kallisto v0.42.3 [26]. As a reference, we used all cDNA sequences from the GRCh38 human genome. Read counts per gene were calculated by summing over their transcripts. Genes with fewer than 10 read counts in at least 2 samples were removed. Sequence data has been deposited in the European Genome-phenome Archive (EGA)—accession number EGAS00001003167.

#### Differential expression analysis

Differential expression analysis was performed using DESeq2 [27] version 1.16.1. The raw RNA-seq counts are modelled as a negative binomial distribution while explicitly normalising for library size. *p* values were adjusted for multiple comparisons using the Benjamini-Hochberg correction (false discovery rate (FDR)).

#### Modular analysis

We applied modular analysis [28, 29] to our RNA-seq data to ask whether any patterns would distinguish the low- and high-episode groups of children (see study population above). We used previously described clusters (modules) of genes that were co-regulated across nine different transcriptomic data sets obtained from patients with a variety of immune conditions [28–32]. Analogous to previously described methods [33], we calculated modular over/underexpression (*s*) as:

$$ {s}_M=100\frac{1}{\mid M\mid}\sum \limits_{i\in M}D\left({g}_{i,a},{g}_{i,b}\right) $$

where

퐷(*g*_*i*, *a*_, *g*_*i*, *b*_)=$$ \left\{\begin{array}{c}\operatorname{sign}\left({\mu}_{i,a}-{\mu}_{i,b}\right)\kern0.5em \mathrm{if}\ p\left({g}_{i,a},{g}_{i,b}\right)<0.05\ \\ {}0\kern0.5em \mathrm{otherwise}\end{array}\right. $$

For each gene *i* within a module *M*, we performed a Mann-Whitney test and calculated the *p* value (*p*) between child groups *a* and *b*. Here, *M* is the set of genes in a module, and |*M*| is the number genes in that module. Child categories include naive, low number of episodes and high number of episodes. If the test yielded a *p* value < 0.05, then the sign of the differences in median rlog values (*μ*) were added to *s*_*M*_ (sign is a function that returns − 1 for negative numbers, 0 for 0, and + 1 for positive numbers). The list of genes in the modules was obtained from a previously published report [29].

In a recent study, a modular transcriptional repertoire analysis was used to find markers for malarial immunity following an RTS,S study [34]. In contrast to modular expression, which describes changes over entire categories of children, we also defined modular response (*r*) for individuals as:

$$ {r}_{c,M}=100\frac{1}{\mid M\mid}\sum \limits_{i\in M}\operatorname{sign}\left({g}_{i,c}-{\mu}_i\right) $$

where *r*_*c,M*_ is the response of child *c* in module *M*, |*M*| is the number of genes in module M, *g*_*i,c*_ is the rlog gene expression of gene *i* in child *c*, and *μ*_*i*_ is the median gene expression of gene *i* in high and low malaria episode children. We then performed a Mann-Whitney test of response rates for each module between high and low malaria episode children.

#### Cellular deconvolution

We performed cellular deconvolution to identify cell-specific gene expression profiles. We learned the gene expression profiles from the LM22 set of genes previously used to deconvolve cell populations from microarray data [35]. To prepare the data for deconvolution, we manually gated cell populations to mirror those used to generate the LM22 gene set. Gene expression was performed on transcripts per million (TPM) as has been previously advocated for RNA-seq measurements [36]. For each gene, we performed deconvolution over seven cell types determined manually as illustrated in Additional file 2: Figure S1 (NK, neutrophil, B cell, CD8^+^ T cell, CD4^+^ T cell, γδ T cell, monocytes) and three child categories (universal (all samples), not naïve (high+low), high). Since deconvolving small populations could be more error-prone, we limited our analysis to the seven cell categories that were present in a significant proportion of the children.

For RNA expression of each gene as measured by TPM, **y**, we fit a profile (**t**) to the fraction of sub-cell types (**F**) measured in children. The sub-cell types are separated into three distinct categories: universal (U), not naive (N) and high episodes (H), and arranged into a matrix as **F** = [**F**(U), **F**(N), **F**(H)]. The universal fraction **F**(U), is the fraction of cells measured for each child. The sum fraction of cells for a child was less than 1, since not all cell events were categorised as a recognisable immune cell. The subsequent terms **F**(N) and **F**(H) are variations of the universal fraction, defined as:

$$ {F}_{c,i}(P)=\left\{\begin{array}{c}{F}_{c,i}(U)\kern0.5em \mathrm{if}\ c\in P\ \\ {}\begin{array}{cc}0& \mathrm{otherwise}\end{array}\end{array}\right. $$

where *P* is a set of children in a category, and *c* is an individual child. We modelled the gene expression as the linear set of equations $\mathbf{Ft}=\widehat{\mathbf{y}}$. For each gene, we fit a profile with lasso penalty as:

$$ {\mathrm{argmin}}_t{\left(\mathbf{Ft}-\mathbf{y}\right)}^T\left(\mathbf{Ft}-\mathbf{y}\right)-\lambda {\left|\mathbf{t}\right|}^1 $$

We chose the lasso penalty (*λ*) that maximised the tenfold cross-validated coefficient of determination (i.e. *R*^2^) to find non-zero cell-specific profiles. This was implemented in Python using scikit-learn [37]. For this lasso penalty, we then performed a Bayesian lasso fit to obtain *z*-scores for the non-zero cell-specific profiles. The model’s parameters were inferred using MCMC [38]. As further controls, we performed this deconvolution on simulated data. In one set, child RNA-seq measurements were scrambled. The resulting number of positive results was used to estimate false discovery rates.

#### Gene set enrichment analysis

Gene set enrichment analysis [39] was performed on the list of genes identified as altered in cell-specific signatures following deconvolution using the Molecular Signatures Database (MSigDB) [40] and queried Gene Ontology terms, Reactome [41, 42] and KEGG [43].

## Results

### Characteristics of study population

Study participants were drawn from two cohorts of children (Junju and Ngerenya) who had been under active surveillance (see the “Materials and methods” section) for 8 years and were selected to fall into 3 categories. Children in the naive group (*n* = 27) had never had clinical malaria, those in the low group (*n* = 21) had experienced less than 5 clinical episodes (median = 2) over the 8-year period and those in the high group (*n* = 21) had a history of 8–18 clinical episodes of malaria (median = 12) (Table 1). There was no discernible difference between the groups in terms of parasitaemia or severity of fever during a clinical episode (Additional file 3: Figure S2). None of the children recruited had experienced a clinical episode within the last 110 days (although unsurprisingly the groups differed in time to the last episode and exposure index), and none were parasitaemic at time of sampling.

**Table 1:** Baseline characteristics of the three epidemiological groups

				*p* value
Town	Ngerenya	Junju		
Group	Malaria-naïve	Low	High	
*n*	27	21	21	
Number of clinical episodes (median [IQR])	0 [0, 0]	2 [1, 2]	12 [9, 14]	< 0.001
Age (mean (sd))	8.8 (1.1)	8.8 (0.3)	8.9 (0.3)	0.9
Sex = M (%)	12 (44.4)	14 (66.7)	9 (42.9)	0.2
Exposure index (median [IQR])	0 [0, 0.02]	0.43 [0.15, 0.57]	0.77 [0.54, 0.81]	< 0.001
Days since last episode (median [IQR])	n/a	490 [380, 1254]	167 [127, 259]	< 0.001
Stool microscopy (%)				0.4
*Ascaris lumbricoides*	0 (0.0)	0 (0.0)	2 (9.5)	
Hook worm	1 (3.7)	1 (4.8)	0 (0.0)	
*Trichuris trichiura*	1 (3.7)	0 (0.0)	0 (0.0)	
No parasites detected	24 (88.9)	20 (95.2)	19 (90.5)	

### Differential gene expression analysis cannot differentiate between naïve and low malaria episode groups

**Fig. 1:**
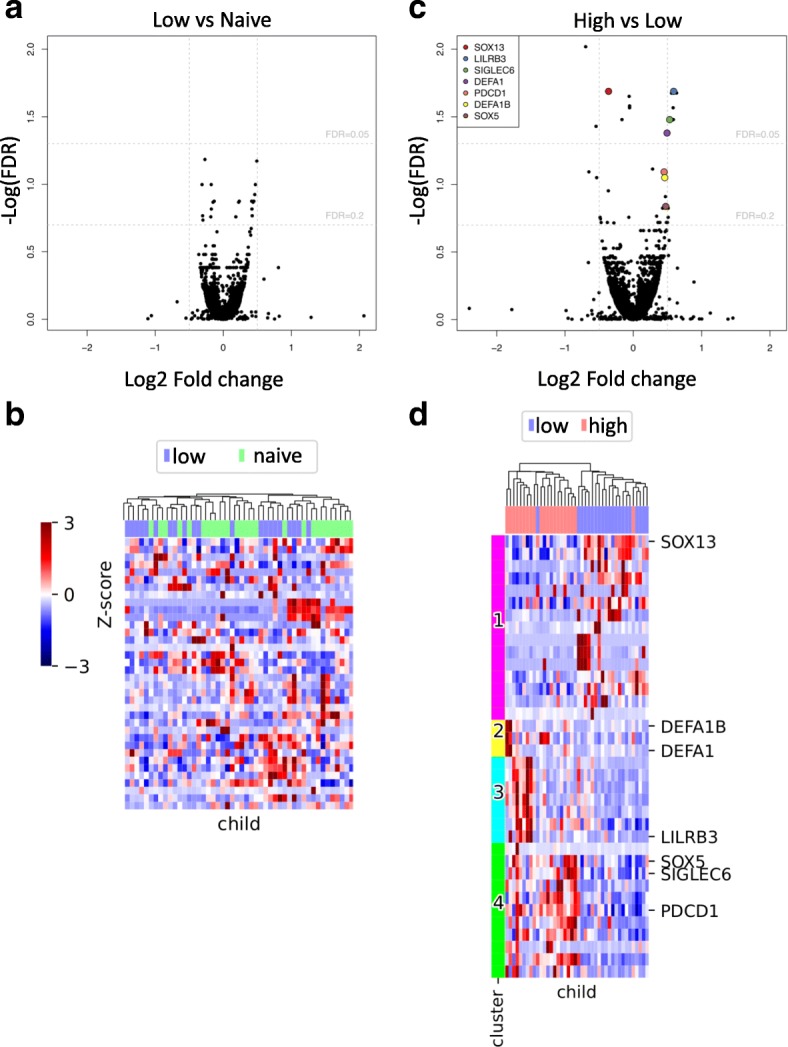
Differential gene expression analysis distinguishes blocks of genes separating high- and low-episode groups. **a** DESeq2 was used to compare the gene expression profiles between naïve and low-episode children. Only small effect sizes were inferred, and most had high FDR values. **b** Hierarchical clustering of individuals based on differentially expressed genes also did not separate individuals into distinct epidemiological groups. **c** Differential gene expression analysis between high- and low-episode children reveals subtle but detectable differences including a number of genes with low FDR and known immunological relevant function (highlighted). **d** We selected 36 gene isoforms with adjusted *p* values < 0.2 as determined by differential gene expression analysis (DESeq2) between low- and high-episode children. We used hierarchical clustering to order children but used *k*-means clustering to identify 4 subsets of gene expression patterns. Child episode category (high/low) are shown for comparison with gene profiles

We compared individuals from the malaria-naïve cohort (Ngerenya) to the individuals who lived in the moderate transmission area of Junju but who had only experienced a low number of cumulative clinical episodes. DESeq2 was used to estimate the group effect size and false discovery rate (FDR) for all genes. Only small effect sizes were inferred, and most had high FDR values (Fig. 1a). Hierarchical clustering of individuals based on genes with an FDR < 0.2 also did not separate individuals into distinct epidemiological groups (Fig. 1b). This shows that we are unable to differentiate between naïve and low-episode individuals on the basis of their transcriptome.

### Differential gene expression analysis differentiates between high and low malaria episode groups

Next, we compared children from the same moderate transmission area but who had experienced low or high numbers of episodes over the 8-year study period. Figure 1c shows the estimated effect size and FDR for all genes. We detected more differentially expressed genes than in the previous analysis of naïve versus low-episode individuals, and while we observe modest effect sizes, there are a small number of genes with low FDR and known immunologically relevant function.

Importantly, hierarchical clustering based on genes with an FDR < 0.2 sorted children almost perfectly into their episode categories. We used *k*-means clustering to identify four gene expression clusters (Fig. 1d; Additional file 4: Table S2). Separation into more than four clusters resulted in additional clusters indistinguishable from one of the first four.

In general, there was quite a high level of heterogeneity within the three groups of children (Additional file 5: Figure S3); however, subtle but detectable differences were observed between the low- and high-episode groups. High-episode individuals appeared to be characterised by a transcriptional signature suggestive of greater immune activation. These clusters feature genes involved in innate immunity, including defensins (such as *DEFA1* and *DEFA1B*) and T cell differentiation (*SOX5*) as well as genes involved in regulating B cell (*SIGLEC6*) and T cell responses (*PDCD1* and *LILRB3*).

### Immune modular analysis reveals a unique signature associated with high number of clinical episodes

**Fig. 2:**
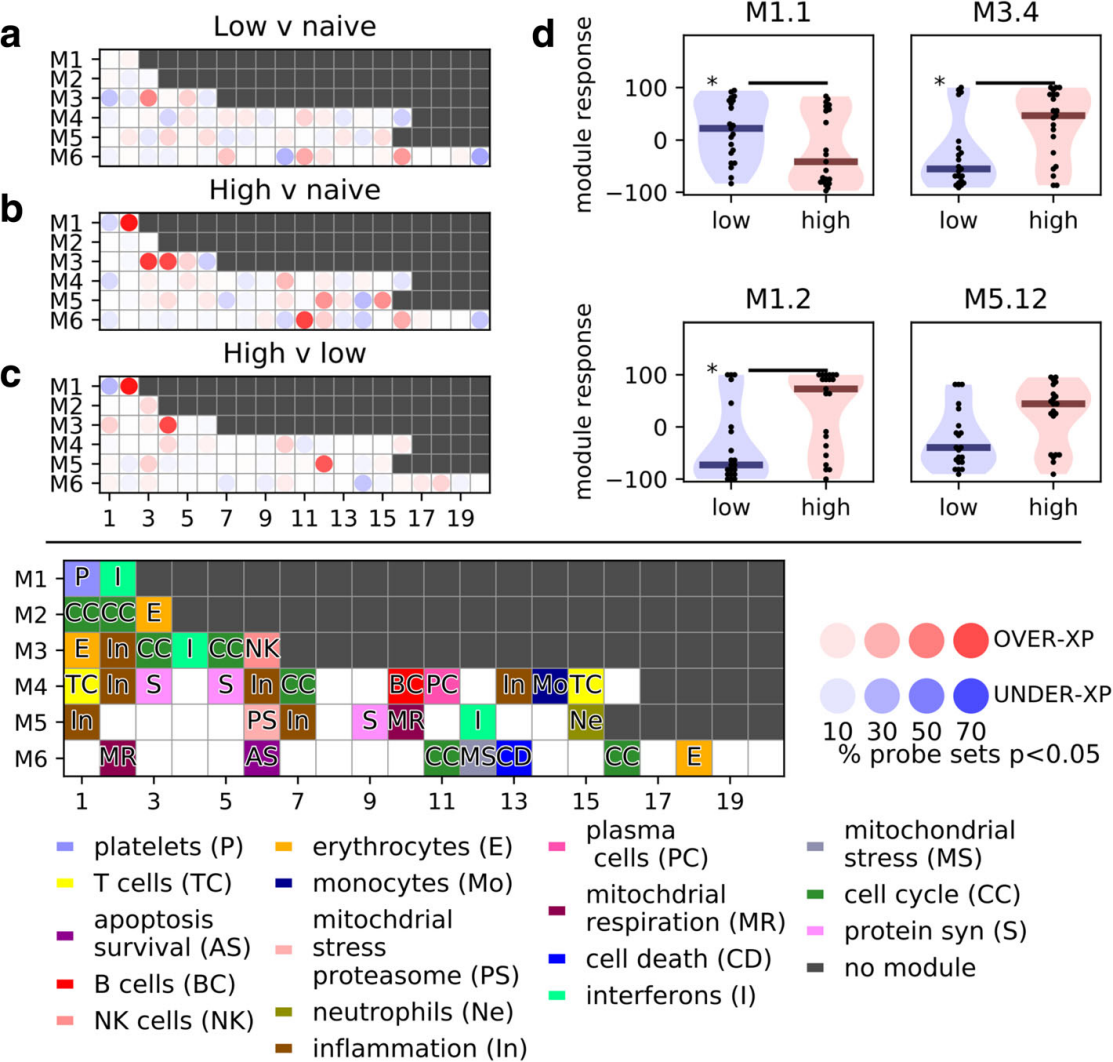
Immune modular analysis reveals a unique signature associated with a high number of clinical episodes. We performed a modular analysis of **a** low-episode versus naive, **b** high-episode versus naive and **c** high-episode versus low-episode children. For each gene within each of these previously defined modules, we performed a Mann-Whitney test between different high-, low-episode, or naive children and determined the number of significant (*p* < 0.05) upregulated and downregulated genes. The overall change in expression is shown as the percentage of up- or downregulated genes, which demonstrates a strong upregulation of the “interferon” modules (M1.2, M3.4, M5.12) in the high-episode group. **d** For each child and each module, we calculated the “modular response” and then performed a Mann-Whitney test of response rates for each module between high and low malaria episode children. Using a Benjamini-Hochberg procedure with FDR cut-off of 20%, we identified three modules that were significantly different between high- and low-episode groups

We applied a modular analysis of immune-related genes [28, 29, 34] to our RNA-seq data to see whether any patterns would distinguish the three groups of children (see the “Materials and methods” section). The overall change in the expression for each module in each epidemiological group is shown in Fig. 2a–c as the percentage of up- or downregulated genes, which demonstrates a strong upregulation of the “interferon” modules (M1.2, M3.4, M5.12) in the high-episode group in line with previous work [34].

To examine the variance in modular expression within the groups, we quantified the modular responses of individual children [34] (Fig. 2d). While there is some heterogeneity within the groups, modules M1.2 and M3.4 (both annotated as “interferon-inducible”) clearly distinguish between the high- and low-episode groups, with both modules expressed more highly in high-episode children. These modules are enriched for genes involved in inflammatory responses and both type I and type II interferon signalling. Similar to the DGE analysis, the modular analysis reveals that high-episode children are characterised by a relative upregulation of immune-related genes within the “interferon-inducible” modules.

### Plasma IL-10 levels are significantly higher in children who have experienced high numbers of clinical episodes

**Fig. 3:**
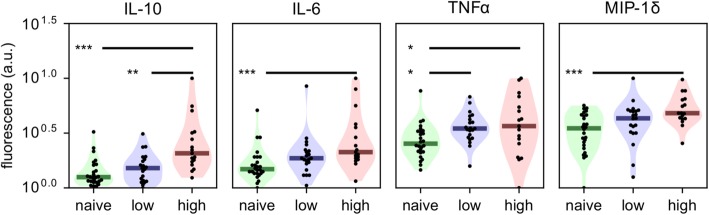
Differences in the levels of cytokines in plasma of naive and low- and high-episode children. Cytokine levels determined by Luminex cytokine array were parameterised as log fluorescence and tested using a three-way Kruskal-Wallis test between naive, low-episode and high-episode groups. Post-hoc Dunn’s tests were performed on cytokines with significant differences. *p = 0.05, **p = 0.01, ***p = 0.005

We next compared plasma cytokine and chemokine levels in the study participants at the time of sampling. Of the 65 cytokines sampled (see the “Materials and methods” section), 30 were below detectable levels. Thirty-one of the remaining cytokines were not significantly different between the groups (Additional file 6: Table S3). We did however find a trend of increasing levels of IL-10, IL-6, TNF-α and CCL15 (MIP-1δ) going from the naïve group to the high group, with IL-10 exhibiting the largest effect (Fig. 3). Indeed, IL-10 levels were significantly higher in children with high numbers of episodes than in low-episode children. Since our modular analysis had revealed a cytokine-inducible gene signature, it was reasonable to suspect associations between the amounts of these plasma cytokines and the modular responses determined above. As expected, plasma levels of IL-10, TNF-α and IL-6, which we found to be higher in the high-episode group were significantly correlated with the “interferon” modules (M1.2, M3.4, M5.12) described above (Additional file 7: Figure S4). These plasma cytokine data therefore provide further evidence of enhanced immune activation and inflammation in the high-episode children and identify elevated levels of the immunoregulatory cytokine IL-10 as a key difference between children who have experienced high and low numbers of episodes.

### Cellular subset composition reveals expansion in activated γδ T cells in high-episode children

In addition to the whole blood transcriptomic analysis, we also characterised the cellular subset composition of the blood samples isolated from each participant by flow cytometry. Using a 14-colour panel of antibodies against a range of human immune cell surface molecules, we determined the relative abundance of the major cell populations in the peripheral blood.

**Fig. 4:**
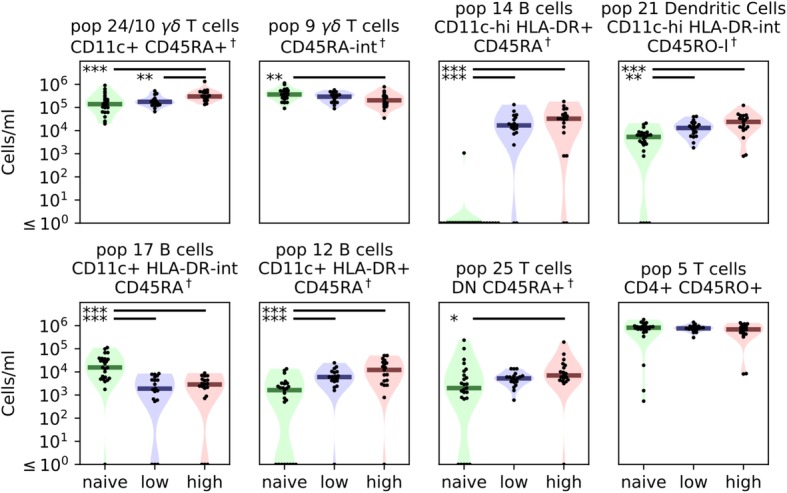
Differences in cellular subset composition of whole blood from naïve and low- and high-episode children. Cellular composition was determined via flow cytometry and analysed as described in the “Materials and methods” section. We used a three-way Kruskal-Wallis test to determine if cell concentrations changed between child categories. We then performed a post-hoc Dunn’s test between individual groups to determine where significant differences occurred. Dendritic cells (population 21) and CD11c^+^ B cells (populations 12, 14 and 17) were clearly able to distinguish between naive and malaria-experienced children. However, we observed only subtle differences between low- and high-episode children with only γδ T cells (merged population 24/10) differing between the two groups. *p = 0.05, **p = 0.01, ***p = 0.005

In addition to classical flow cytometric analysis using manual gating, we maximised the objectivity and descriptive power of our analysis by using Cytofkit [23] to perform an unsupervised analysis of our FACS data. Cellular subsets were identified using a clustering algorithm and individual populations annotated using a heatmap displaying the median intensity values per cluster for every marker. This method identified 25 populations (Additional file 8: Figure S5), which were further curated manually. Merging biologically indistinguishable populations and excluding unidentifiable populations resulted in 15 identifiable cellular populations (Additional file 9: Figure S6). This analysis revealed that cellular subset composition is significantly associated with malaria experience. The numbers of CD11c^+^ B cells (populations 12, 14 and 17), γδ T cells (populations 9, 24/10), double-negative T cells (population 25) and dendritic cells (population 21) in naïve individuals were significantly different from those in either of the other two groups (Fig. 4). CD11c^hi^ B cells (population 14) in particular are practically absent from the naive group while found at levels over 10^4^ cells/mL in the other groups. CD11c^+^ γδ T cells were significantly expanded in the high-episode group and were the only population of those we characterised to distinguish between the high- and low-episode groups.

### Children with high numbers of episodes show transcriptionally altered CD8^+^ T cells, B cells and neutrophils

**Fig. 5:**
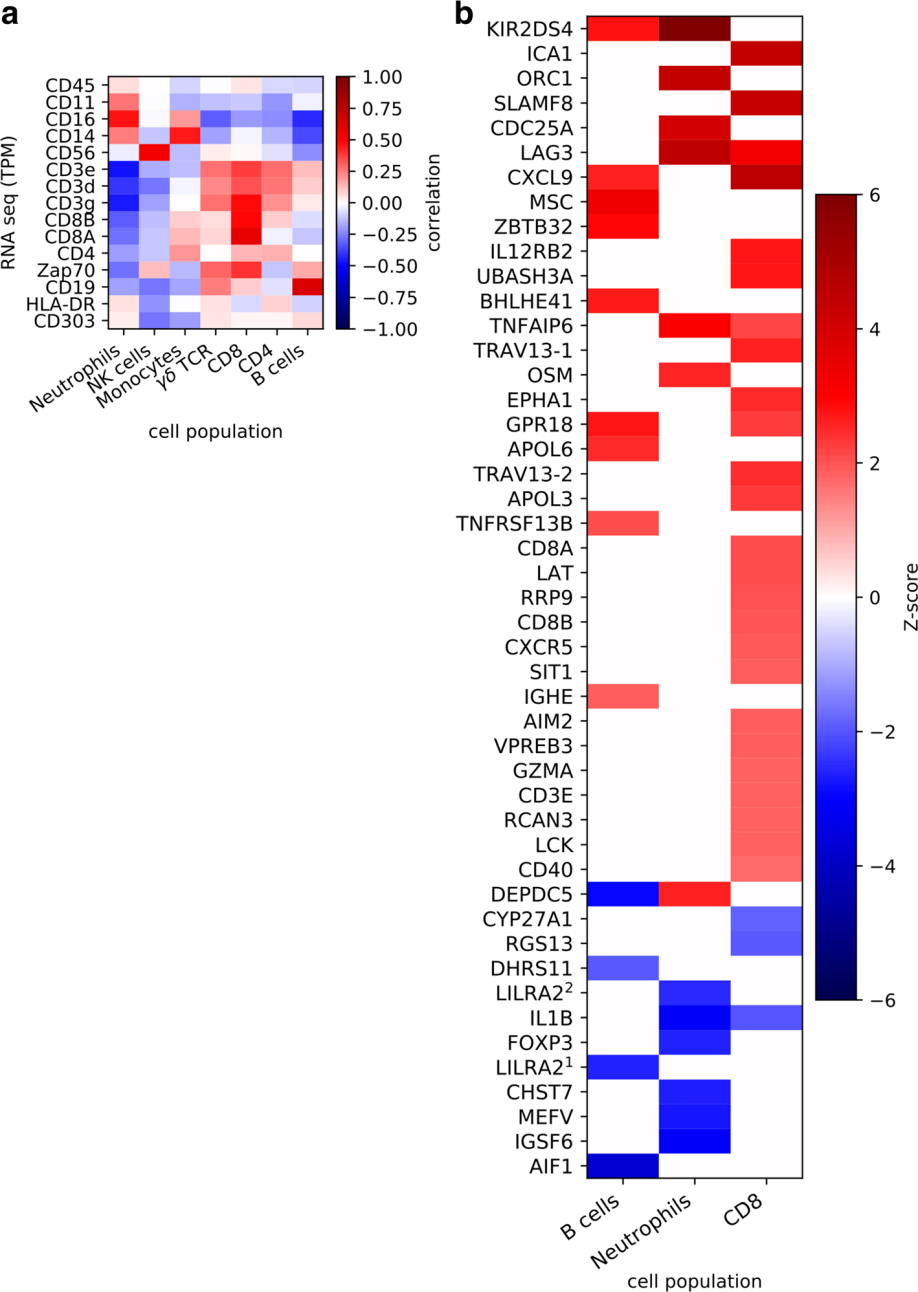
Cell-specific gene expression profiles inferred from cellular deconvolution of transcriptome reveal transcriptionally altered CD8^+^ T cells, neutrophils and B cells in high-episode children. **a** RNA expression of canonical cellular lineage-associated markers strongly correlated with inferred cell-specific profiles, suggesting that cell-specific gene expression patterns can be successfully deconvolved from cell sub-type proportions and RNA levels. **b** Using scrambled data to infer false discovery rate, cell-specific gene contributions were selected. Focusing on cell populations with at least ten genes altered, high-episode children show transcriptionally altered CD8^+^ T cells, neutrophils and B cells. Heatmap illustrates the expression level of cell-specific genes in high-episode children relative to low-episode children

A major caveat of whole blood transcriptomic analysis is that observed differences in transcript levels might represent changes in the abundance of certain cellular populations but not necessarily changes in gene expression within individual cell populations [44]. We therefore performed cellular deconvolutions where cell-specific gene expression profiles were inferred based on FACS measurements of cell proportions and RNA-seq transcript levels. To validate the method, we first demonstrated that RNA expression of canonical lineage-associated markers associated well with the inferred cell profiles and found good correlations between each inferred subset and its respective subset associated marker (Fig. 5a).

As cellular deconvolution can be confounded by the sensitivity of smaller populations to changes in larger subsets, we limited the analysis to the populations that accounted for 10% or more of the total cell population. Three populations (CD8^+^ T cells, B cells and neutrophils) demonstrated a transcriptionally altered phenotype with ten or more altered genes in high-episode children relative to low-episode children (Fig. 5b). The majority of immunologically relevant genes upregulated in high-episode children were associated with CD8^+^ T cells. Gene set enrichment analysis (GSEA) on the significantly altered genes for these cells showed enrichment for genes involved in positive activation of lymphocytes (including *LAT*, *LCK* and *CD40*), which could suggest more active CD8^+^ T cells in high-episode children (Additional file 10: Table S4). The B cell deconvolution profile also features mostly upregulated gene sets in this group of children. Many of these genes have been implicated in B cell receptor signalling and regulating antibody responses (and include *TNFRSF13B*, *ZBTB32* and *MSC*). B cells from high-episode children also expressed higher levels of *IGHE* (gene encoding IgE). Neutrophils in high-episode children were associated with the upregulation of genes involved in the defence and inflammatory responses including *OSM* and *TNFAIP6*. Our deconvolution approach thus suggests that high-episode children are distinguished by transcriptionally altered CD8^+^ T cells, B cells and neutrophils, characterised by the upregulation of key immune-related genes.

## Discussion

In this multi-dimensional assessment of the association between repeated malaria infections and immune phenotype, we combined data from whole blood transcriptomic analysis, multi-parameter flow cytometry, multiplex plasma cytokine analysis and active malaria surveillance to identify the immunological features associated with clinical malaria experience. We observed subtle but detectable differences in gene expression between children who have experienced a high number of episodes compared with others who have experienced fewer episodes. High-episode children were associated with increased expression of genes involved in immune activation and regulation, with modular analysis revealing the enrichment in genes involved in responses to type I and II interferons. The transcriptomic signature of enhanced immune activation in high-episode children is supported by our findings that levels of IL-10 and numbers of a subset of γδ T cells are significantly higher in these children compared to low-episode children. Through cellular deconvolution of the transcriptomic data, we found that high-episode children may have transcriptionally altered CD8^+^ T cells, B cells and neutrophils.

Notably, we observed a modular transcriptional signature that differs between high- and low-episode children. High-episode children were characterised by higher expression of three modules containing interferon-inducible genes. These three modules (M1.2, M3.4 and M5.12) are part of the transcriptional signature associated with protection of malaria-naïve adults following the administration of the RTS,S malaria vaccine [34]. They have also been shown to become sequentially activated in systemic lupus erythematosus (SLE) patients [30] and form part of the transcriptional signature associated with the trivalent influenza vaccine [29]. While module M1.2 is enriched for genes induced by IFN-α, modules M3.4 and M5.12 are capable of also being driven by IFN-β and IFN-γ [30]. This appears to suggest a role for both type I and type II interferons in shaping the immune system within high-episode individuals. Cellular immunity to malaria is typically thought to involve IFN-γ produced by Th1 CD4^+^ T cells; however, both type I and II interferons have been implicated in the immune response to malaria. Type I interferons, produced by a number of cell types following malaria infection [31, 32, 45–47], have been implicated in regulating CD4^+^ T cell responses and promoting the differentiation of IL-10-producing Tr1 cells [48], which are known to be significantly expanded in highly exposed children [49]. This immunoregulation is thought to reflect an attempt by the immune system to limit inflammation-induced immunopathology but comes at the cost of limiting anti-parasite immunity and may interfere with the induction of robust vaccine-induced immunity.

Inflammatory innate and adaptive immune responses are crucial for parasite clearance; however, these effector functions can result in significant immunopathology without appropriate regulation [50, 51]. IL-10 plays a crucial role in modulating the inflammatory response during malaria [51], and it is notable that even in non-parasitaemic children, of the 65 cytokines measured in plasma, IL-10 was the only cytokine observed at significantly different levels between high- and low-episode individuals. While a number of different cell types produce IL-10, a major source in malaria infections is CD4^+^ T cells that co-produce IFN-γ and IL-10 (Tr1 cells) [49]. These cells are prevalent in children living in endemic areas, and the IL-10 they produce has been shown to inhibit malaria-specific pro-inflammatory cytokine production [52]. Though we do not address the cellular source of IL-10 in this study, we found that increased plasma levels of IL-10 were significantly associated with increased expression of the “interferon-inducible” signature in high-episode children, in keeping with the known role of interferons in inducing the development of Tr1 cells [48].

At the cellular level, a subset of γδ T cells (population 24/10) was significantly expanded in high- relative to low-episode children. γδ T cells are activated during malaria, but their function in anti-malarial immunity remains unclear. These cells have been shown to expand during acute malaria infection in previously naïve individuals [53, 54] and can produce inflammatory cytokines including TNF-α and IFN-γ [55] in addition to being able to directly kill merozoites in vitro [56, 57]. More recently, a subset of γδ T cells has been shown to associate with the protection in irradiated sporozoite vaccination [58]. In this study, we found that a specific subset of γδ T cells, expressing CD11c, accumulates in high-episode children. While not previously reported in the context of malaria, CD11c^+^ γδ T cells have been described as a highly activated subset with enhanced effector function and high migratory potential [59].

Deconvolution analysis integrating cellular proportions (as determined by flow cytometry) with transcriptomic data allowed us to infer altered gene expression profiles in neutrophils, CD8^+^ T cells and B cells in high-episode children. Very little is known about the role of neutrophils in malaria although neutrophils isolated from *Pf*-infected children in the Gambia were shown to temporarily exhibit reduced effector function until about 8 weeks after infection [60]. Our results suggest that repeated episodes of malaria result in the development of an activated neutrophil phenotype that persists even in the absence of detectable infection.

Our finding of high levels of B cell expression of genes including TNF receptor superfamily member 13B (*TNFRSF13B*), a receptor found on the surface of B cells, responsible for regulating humoral responses and survival of plasma cells [61], is in line with the studies demonstrating that repeated exposure to malaria is necessary for the development of appropriate humoral responses [3–5]. Furthermore, our finding that B cells from high-episode children also express high levels of IgE supports previous studies showing increased plasma IgE levels in individuals living in high transmission settings [62, 63]. We also revealed a clear expansion of CD11c^+^ B cells in malaria-experienced children. Atypical B cells (a population that includes CD11c^+^ B cells) have previously been identified in high frequencies among individuals living in malaria-endemic regions [9]. While these cells were completely missing in naïve children, we did not observe significant differences in CD11c^+^ B cell numbers between the high- and low-episode groups. This suggests that although malaria most certainly leads to the initial expansion of these cells, they may not accumulate with subsequent episodes.

More unexpected was our finding that increased malaria experience results in more activated CD8^+^ T cells. CD8^+^ T cells have clear roles in the immune response to pre-erythrocytic stages of infection [64, 65] and have been implicated in mediating pathology in a murine model of cerebral malaria [66, 67]. There is evidence that CD8^+^ T cells specific to blood-stage antigens are activated via cross-presentation by dendritic cells [68] and may indirectly promote immunity through secretion of IFN-γ [11, 69]. It is interesting to note that a recent study in Western Kenya has described the expansion of an unconventional innate-like CD8^+^ T cell population in children living in an area of high parasite burden [70]. While future studies will be needed to confirm the presence of this cellular subset among our cohort, this study provides further evidence of transcriptional alteration of CD8^+^ T cells in the context of malaria exposure.

In our study, participants were selected on the basis of numbers of preceding episodes of malaria within the past 8 years; however, it is important to note that unsurprisingly from an epidemiological standpoint, these children also differ in two other important aspects. Despite the fact that none of the participants in either group had experienced an episode of malaria for more than 110 days, there was a significant difference in the calculated exposure indices and time to the last episode between the low- and high-episode groups. While the modular transcriptional signature we observed does not appear to correlate with time to the last episode (Spearman correlations: M1.1 = 0.027, *p* value = 0.87; M1.2 = − 0.05, *p* value = 0.76; M3.4 = − 0.08, *p* value = 0.63; M5.12 = − 0.13, *p* value = 0.43), we cannot discount the possibility that the effects that we observe are due in part at least to the more recent immunological stimulation in the high-episode group rather than the number of previous episodes per se. Carefully designed longitudinal studies would be required to disentangle the contributions of these and other parameters to the development of a malaria immune response. These studies could prospectively relate individual pre-existing immunological status to subsequent risk of clinical infection and thus determine which immune responses are directly related to clinical protection.

## Conclusion

In summary, in this exploratory study, we show that our approaches of transcriptomic analysis together with cytokine and immune cell profiling of peripheral blood can robustly detect immune differences between high- and low-episode children. Multiple (and possibly recent) episodes of malaria are associated with the modification of the immune system in children. Individuals who have experienced repeated episodes demonstrate enhanced activation of neutrophils, B cells and CD8^+^ T cells; upregulation of interferon-inducible genes; and a clear increase in circulating levels of the immunoregulatory cytokine IL-10. Such elevated IL-10 levels suggest a degree of immune modulation that may be important for avoiding immunopathology but could interfere with parasite clearance. This skewing may also affect the induction of protective immune responses by vaccines and hence have significant implications for the efficacy of such vaccines in endemic populations.

## Additional files

Additional file 1: **Table S1.** Flow cytometry antibody panel. (XLSX 9 kb)

Additional file 2: **Figure S1.** Gating strategy used to define cellular subsets used in deconvolution analysis. (PDF 425 kb)

Additional file 3: **Figure S2.** Distribution of temperature and log-parasitaemia for each clinical episode in the high- and low-episode groups over period of follow-up. High-episode group: red dots, *n* = 21; low-episode groups: blue dots, *n* = 21 (PDF 160 kb)

Additional file 4: **Table S2.** *k*-means clustering of differentially expressed genes between low- and high-episode children. (XLSX 10 kb)

Additional file 5: **Figure S3.** Principal component analysis (PCA) plot of the transcriptome profiles of study participants. Naïve (green), low episodes (blue) and high episodes (red). (PDF 39 kb)

Additional file 6: **Table S3.** Mean levels of detectable plasma cytokines. (XLSX 11 kb)

Additional file 7: **Figure S4.** Association between immune modular expression and plasma cytokine levels. (a) Spearman correlations between significant cytokines and modules are shown along with (b) their respective *p* values. (PDF 278 kb)

Additional file 8: **Figure S5.** Unsupervised cellular subset identification. Flow cytometry data was analysed using the integrated analysis pipeline Cytofkit. (a) Collective t-SNE dimensionality reduced CD45^+^ live cell data derived from 69 participants. Every dot represents a single cell, and the colour of the cells indicates the expression values for a given marker analysed. (b) Cellular subsets were identified using Cluster X. (c) Heatmap displaying hierarchical clustering of median surface marker expression levels of indicated populations. Bracketed clusters were condensed into one population. (Populations 13, 7, 18, 19 and 15 determined to be unidentifiable). (PDF 1238 kb)

Additional file 9: **Figure S6.** Cellular composition of whole blood from naïve and low- and high-episode children. The initial clusters in Additional file 8: Figure S5 were manually curated, merging biologically indistinguishable clusters resulting in 15 identifiable cellular populations. We used a 3-way Kruskal-Wallis test to determine if cell concentrations changed between child categories. We then performed a post-hoc Dunn’s test between individual groups to determine where significant differences occurred. *p = 0.05, **p = 0.01, ***p = 0.005. (PDF 781 kb)

Additional file 10: **Table S4.** Results of GSEA analysis of CD8^+^ T cell deconvolution signature associated with high-episode children. (XLSX 13 kb)
