# Supplementary figures and images for: Repeated clinical malaria episodes are associated with modification of the immune system in children
Source: BMC Med. 2019 Mar 13;17:60. doi: 10.1186/s12916-019-1292-y (PMC6415347; doi:10.1186/s12916-019-1292-y)

# Supplementary Figure S1

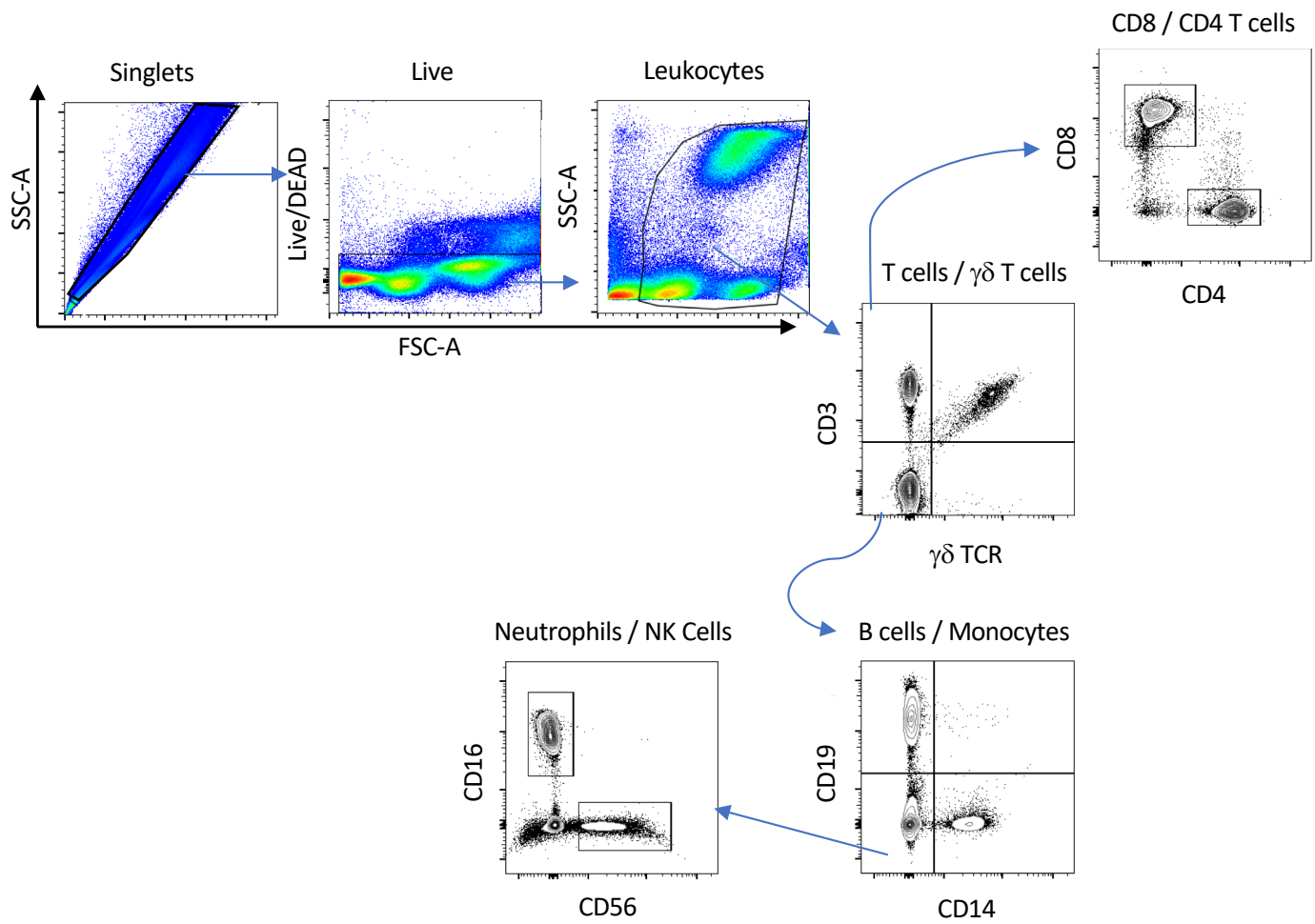

Supplement: Supplementary file 2 — Figure S1. Gating strategy used to define cellular subsets used in deconvolution analysis. (PDF 425 kb) [file 12916_2019_1292_MOESM2_ESM.pdf]

Supplementary Figure S2

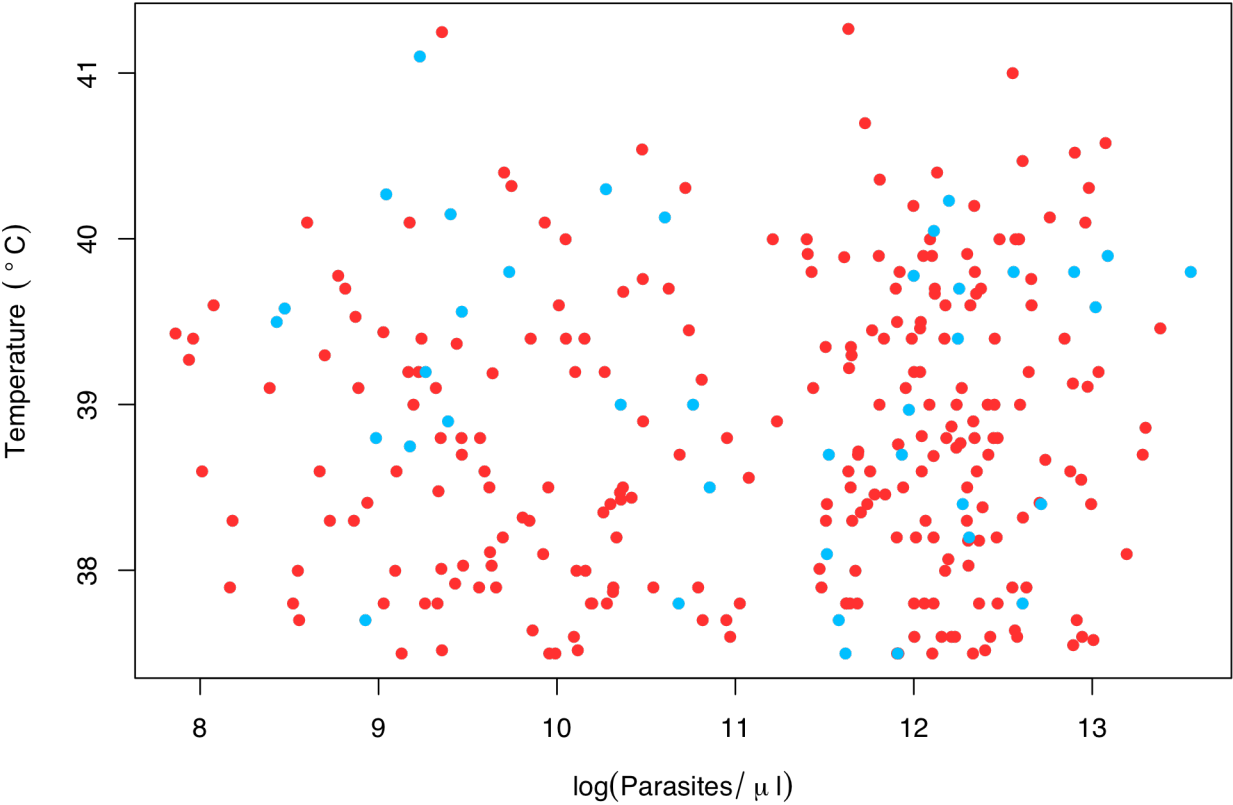

Supplement: Supplementary file 3 — Figure S2. Distribution of temperature and log-parasitaemia for each clinical episode in the high- and low-episode groups over period of follow-up. High-episode group: red dots, n = 21; low-episode groups: blue dots, n = 21 (PDF 160 kb) [file 12916_2019_1292_MOESM3_ESM.pdf]

# Supplementary Figure S3

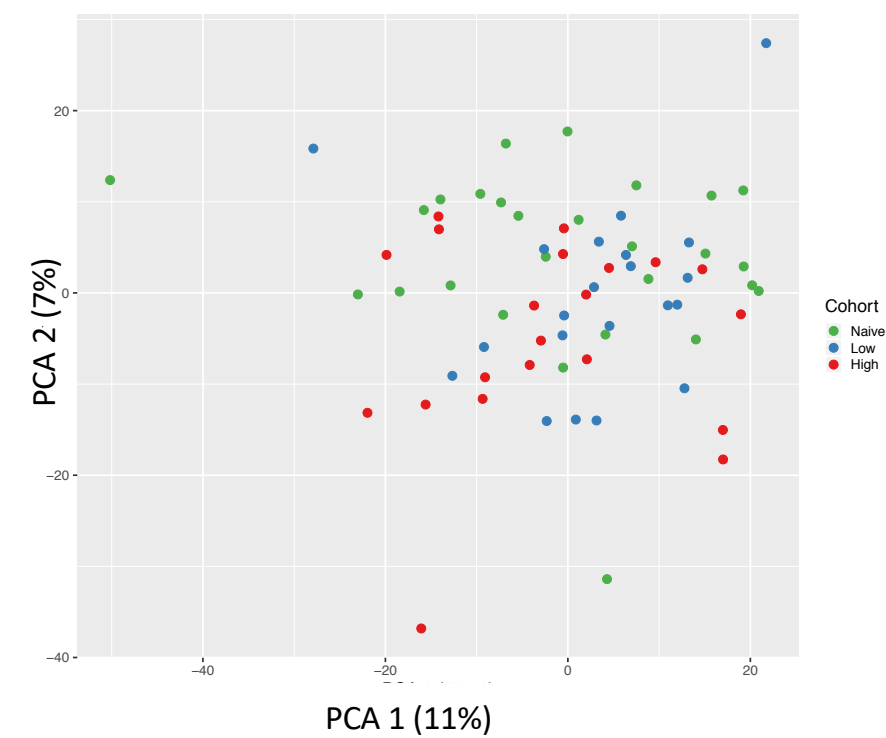

Supplement: Supplementary file 5 — Figure S3. Principal component analysis (PCA) plot of the transcriptome profiles of study participants. Naïve (green), low episodes (blue) and high episodes (red). (PDF 39 kb) [file 12916_2019_1292_MOESM5_ESM.pdf]

# Supplementary Figure S4

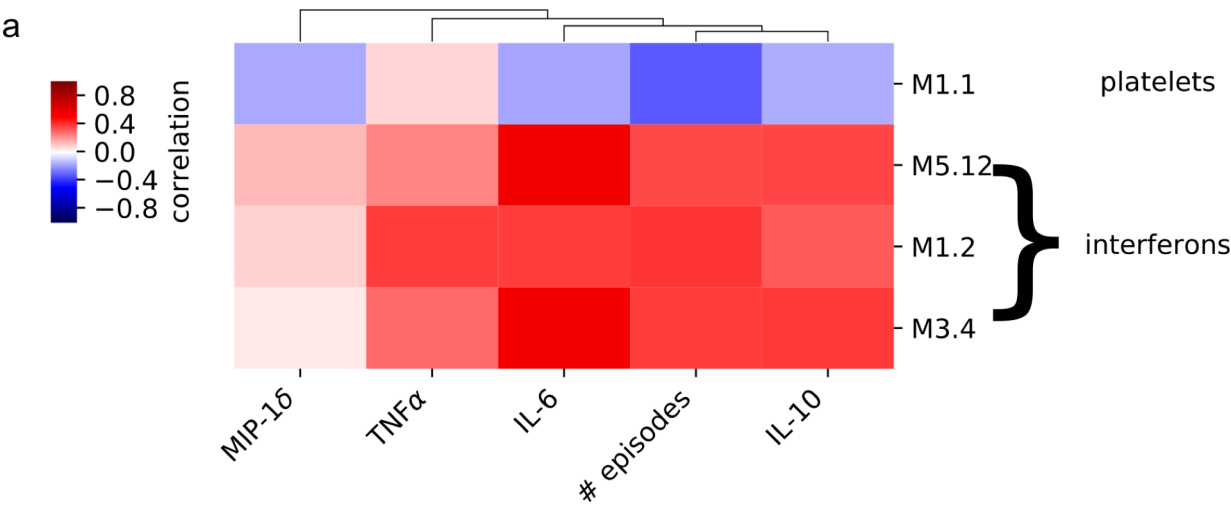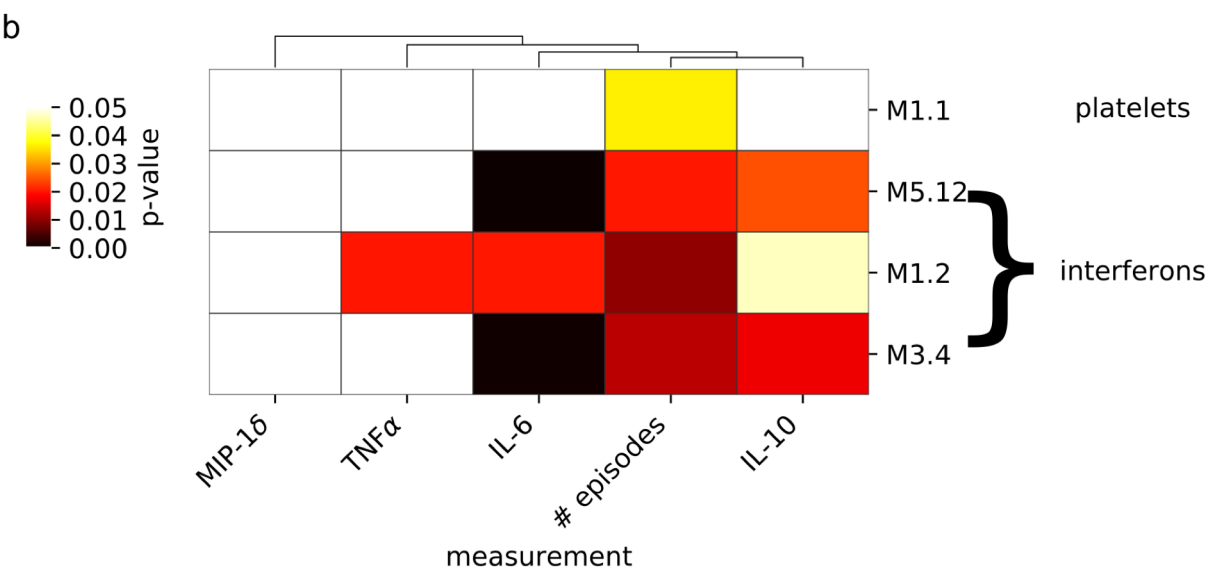

Supplement: Supplementary file 7 — Figure S4. Association between immune modular expression and plasma cytokine levels. (a) Spearman correlations between significant cytokines and modules are shown along with (b) their respective p values. (PDF 278 kb) [file 12916_2019_1292_MOESM7_ESM.pdf]

# Supplementary Figure S5

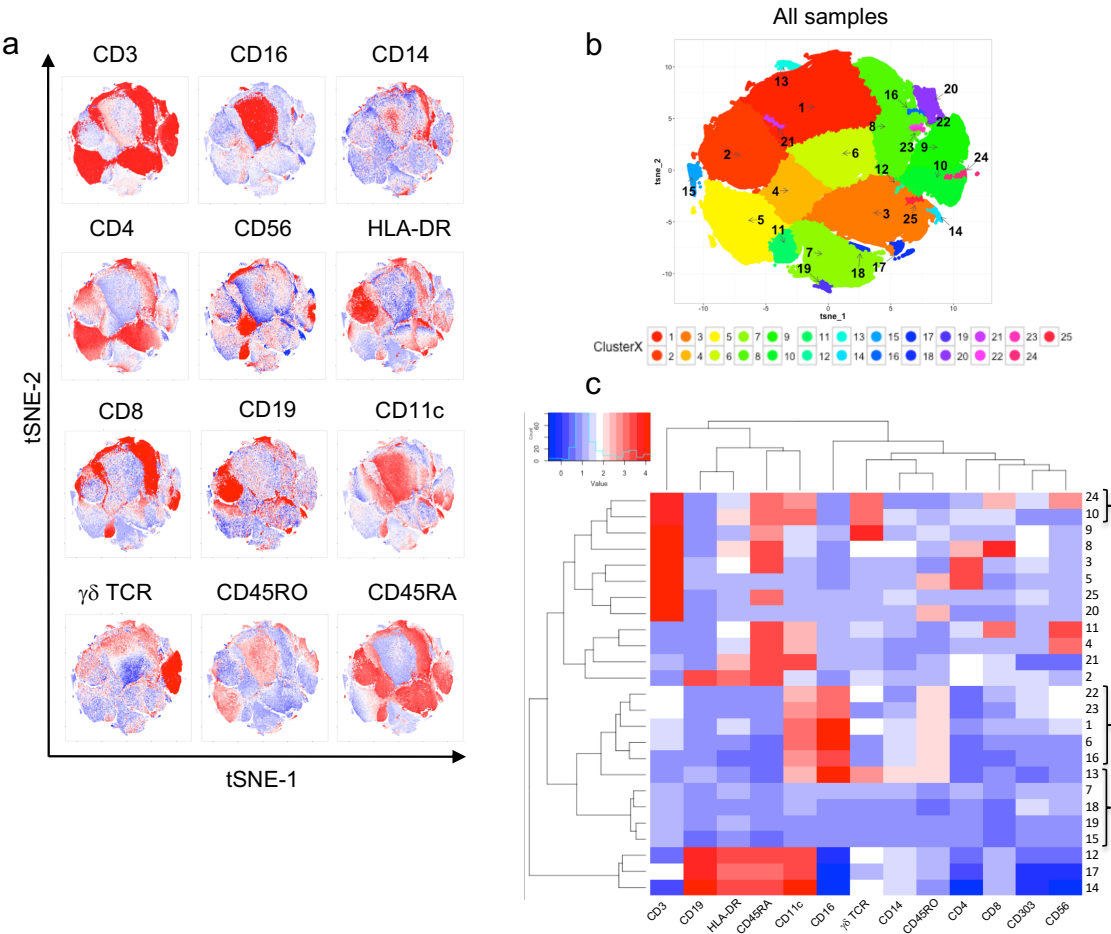

Supplement: Supplementary file 8 — Figure S5. Unsupervised cellular subset identification. Flow cytometry data was analysed using the integrated analysis pipeline Cytofkit. (a) Collective t-SNE dimensionality reduced CD45+ live cell data derived from 69 participants. Every dot represents a single cell, and the colour of the cells indicates the expression values for a given marker analysed. (b) Cellular subsets were identified using Cluster X. (c) Heatmap displaying hierarchical clustering of median surface marker expression levels of indicated populations. Bracketed clusters were condensed into one population. (Populations 13, 7, 18, 19 and 15 determined to be unidentifiable). (PDF 1238 kb) [file 12916_2019_1292_MOESM8_ESM.pdf]

# Supplementary Figure S6

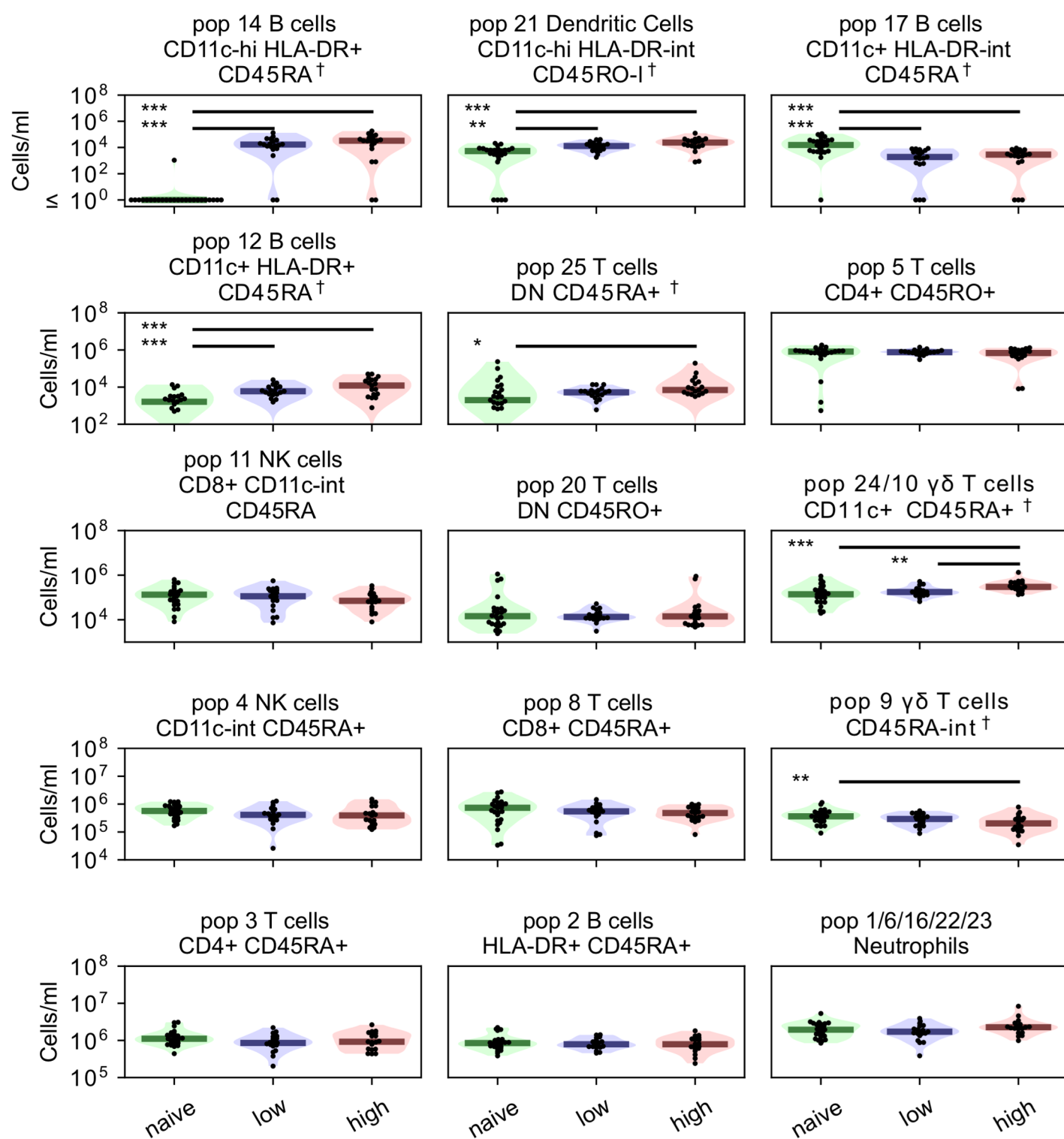

Supplement: Supplementary file 9 — Figure S6. Cellular composition of whole blood from naïve and low- and high-episode children. The initial clusters in Additional file 8: Figure S5 were manually curated, merging biologically indistinguishable clusters resulting in 15 identifiable cellular populations. We used a 3-way Kruskal-Wallis test to determine if cell concentrations changed between child categories. We then performed a post-hoc Dunn’s test between individual groups to determine where significant differences occurred. *p = 0.05, **p = 0.01, ***p = 0.005. (PDF 781 kb) [file 12916_2019_1292_MOESM9_ESM.pdf]
